# *Lycium barbarum* and *Lactobacillus acidophilus* synergistically protect against anti-tuberculosis drug-induced male reproductive injury via gut microbiota-independent pathways in mice

**DOI:** 10.3389/fmicb.2025.1622017

**Published:** 2025-07-21

**Authors:** Xiaoyong Song, Wei Guan, Zhimin Du, Yi Gong, Dan Wang, Yajun Xiong, Yuting Gao, Xinli Shi

**Affiliations:** ^1^Laboratory of Integrated Medicine Tumor Immunology, Shanxi University of Chinese Medicine, Taiyuan, China; ^2^The First Clinical Medical College, Beijing University of Chinese Medicine, Beijing, China; ^3^Department of Andrology, Dongzhimen Hospital, Beijing University of Chinese Medicine, Beijing, China

**Keywords:** *Lycium barbarum*, gut microbiota, male reproductive injury, intestinal barrier, *Lactobacillus acidophilus*

## Abstract

**Background:**

As first-line anti-tuberculosis drugs, rifampicin (RIF) and isoniazid (INH) are associated with reproductive impairment during their use, accompanied by sustained dysbiosis of the gut microbiota (GM). *Lycium barbarum* (Wolfberry), a substance that can be used both as medicine and food, is often used in traditional Chinese medicine to treat male reproductive-related diseases. However, the potential of wolfberry to mitigate reproductive injury induced by anti-tuberculosis (anti-TB) drugs via modulation of the GM has not been reported. This study aimed to explore the protective effect and mechanism of wolfberry on the reproductive injury of male mice induced by anti-TB drugs.

**Methods:**

Forty male Kunming mice were randomly assigned to normal, model, wolfberry, and levocarnitine groups (*n* = 10/group). The normal group received a daily gavage of ultrapure water, while the other three groups were administered ultrapure water, wolfberry decoction, and levocarnitine, respectively, via gavage 3 h prior to the daily administration of RIF and INH for 21 days. Another 40 mice were rendered pseudo-germ-free via oral administration of antibiotic (ATB) water for 1 week, then divided into ATB, ATB + Wolfberry, ATB + *Lactobacillus acidophilus* (*L. acidophilus*), and ATB + Wolfberry+*L. acidophilus* groups. Prior to the administration of RIF and INH by gavage, the mice were administered ultrapure water, wolfberry decoction, *L. acidophilus*, or a combination of wolfberry and *L. acidophilus* via gavage for 21 consecutive days. Afterwards, sperm motility, count, and serum follicle-stimulating hormone (FSH), luteinizing hormone (LH), and testosterone (T) levels were evaluated. Gut contents were collected for 16S rRNA sequencing and real-time PCR, and testicular tissues were subjected to pathological and transcriptomic analyses.

**Results:**

Wolfberry improved sperm quality in mice with reproductive injury induced by anti-TB drugs. Specifically, wolfberry increased sperm count and motility, alleviated testicular pathological damage, and regulated the levels of sex hormones, including FSH, LH, and T. Besides, wolfberry restored intestinal barrier function, enhanced the abundance of *L. acidophilus* in the gut, and modulated key processes involved in spermatid differentiation, sperm development, and the meiotic cell cycle. Notably, the combination of wolfberry and *L. acidophilus* yielded the most significant protective effects against reproductive injury induced by anti-TB drugs.

**Conclusion:**

Our findings suggest that wolfberry protects against reproductive injury induced by anti-TB drugs, partially mediated through modulation of the GM, though this effect is not entirely dependent on the microbiota. Importantly, wolfberry and *L. acidophilus* play a synergistic role in protecting against the reproductive injury induced by anti-TB drugs.

## 1 Introduction

Tuberculosis (TB) remains a major global public health issue, with around 55% of those affected being male (World Health Organization, 2024; Chihota et al., 2025). Once diagnosed, TB patients typically require long-term medication, which often involves a combination of anti-tuberculosis (anti-TB) drugs (Suárez et al., 2019). However, the utilization of anti-TB medications is linked to various side effects, notably impacting male reproductive health. These effects may include abnormal sperm morphology, histological damage to the testes, reproductive toxicity, and a reduction in male fertility and fertilization ability (Shayakhmetova et al., 2012; Awodele et al., 2016; Bakare et al., 2022). Thus, the identification of effective protective strategies to mitigate the adverse impacts of anti-TB medications on the male reproductive system is crucial.

*Lycium barbarum* (Wolfberry), known as a traditional herbal medicine, has been subject to extensive research over the past decade for its potential health benefits, especially concerning reproductive health and antioxidant effects (Shi et al., 2017). In clinical practice, wolfberry and its compound preparations have been widely used in the treatment of male reproductive injuries (Wang et al., 2021). Recent studies have suggested that wolfberry may protect against various forms of oxidative stress and inflammation, including those induced by pharmaceutical treatments (Cao et al., 2025; Ma et al., 2025). Wolfberry contains a range of bioactive compounds such as polysaccharides, flavonoids, and carotenoids, which are thought to improve cellular health, enhance immune function, and protect tissues from damage (Yu et al., 2023). A substantial body of research has confirmed that the extracts of wolfberry can enhance testicular function, promote testosterone synthesis, and improve sperm quality (Qian, 2019; Liang et al., 2025). However, its specific role in protecting against reproductive injury induced by anti-TB drugs is yet to be fully elucidated.

The gut microbiota (GM) has become recognized as a key factor in maintaining a range of physiological processes, including reproduction. Gut dysbiosis is now understood to result in systemic inflammation and impact reproductive health (Cai et al., 2022; Chen et al., 2024; Wei et al., 2024). Recent studies have demonstrated that long-term gut dysbiosis induced by anti-TB drugs may represent a significant contributing factor to the impairment of male reproductive health (Namasivayam et al., 2017; Cao et al., 2021). However, the role of the GM in facilitating the protective benefits of wolfberry in this context remains to be elucidated. Furthermore, it is essential to explore whether wolfberry exerts its beneficial effects through its impact on the GM or through other mechanisms, such as direct modulation of spermatogenesis and testicular health.

This study aimed to investigate the protective effects of wolfberry against male reproductive injury induced by anti-TB drugs, with emphasis on its potential to restore sperm quality, testicular structure, and GM. We also explored whether the GM was involved in these protective effects and examined the synergistic interaction between wolfberry and *Lactobacillus acidophilus* (*L. acidophilus*) to enhance its therapeutic efficacy.

## 2 Materials and methods

### 2.1 Experimental animals and ethical approval

Specific pathogen-free (SPF)-grade, six-week-old male Kunming (KM) mice [SCXK (Jing) 2019-0010, SiPeiFu, China] were housed in a barrier facility at Shanxi University of Chinese Medicine under controlled conditions: temperature of 23 ± 1°C, relative humidity of 50 ± 10%, and a 12-h light–dark cycle. The mice had unrestricted access to food and water. Before the experiment, the animals underwent a seven-day acclimatization period to reduce stress from transportation. The study was approved by the Animal Experimental Ethics Committee of Shanxi University of Chinese Medicine (Approval No.: AWE202407364) and followed the “3R principles” throughout the experimental procedure.

### 2.2 Drugs and chemicals

The dried wolfberry (2210038, TongRenTang Chinese Medicine, China) fruits were soaked in ultrapure water for 30 min, first boiled with high heat, then decocted with low heat for 30 min, and the wolfberry decoction was collected by filtration. The filter residue was added with ultrapure water and decocted again with low heat for 30 min. The two filtrates were combined and concentrated, then dispensed and stored in an ultra-low temperature refrigerator. Before use, the wolfberry decoction should be heated to 37°C. Rifampicin (R8011, Solarbio, China) and isoniazid (I8460, Solarbio, China) were dissolved in a 0.5% sodium carboxymethylcellulose solution prior to use. Levocarnitine (H19990372, NOETHEAST PHARM, China) was also prepared for the experiment. The *L. acidophilus* used in this study was cultured independently by the research team. The antibiotic (ATB) water was prepared with 5 mg/mL streptomycin (3810-74-0, MACKLIN, China), 1 mg/mL ampicillin (69-52-3, MACKLIN, China), and 0.25 mg/mL vancomycin (1404-93-9, PUKE, China). This formulation was based on a previous study conducted by our research group and was added to the daily drinking water of the mice (Liu et al., 2023).

### 2.3 Grouping and intervention

First, 40 male KM mice were randomly assigned to four groups: the normal group, the model group, the wolfberry group, and the levocarnitine group (*n* = 10/group). The normal group and the model group were administered daily gavage of ultrapure water (0.2 mL/d). The wolfberry group and the levocarnitine group received wolfberry decoction (1.75 g/kg/d) and levocarnitine (28 mg/kg/d), respectively, also via gavage. Three hours later, the model group, the wolfberry group, and the levocarnitine group were all given a suspension containing rifampicin (100 mg/kg/d) and isoniazid (50 mg/kg/d) via gavage (Bai et al., 2022). This intervention lasted for 21 days.

Besides, another cohort of 40 KM mice was first provided with ATB water for 7 days to establish a pseudo-germ-free mouse model. Subsequently, these mice were randomly divided into four groups: the ATB group, the ATB + Wolfberry group, the ATB + *L. acidophilus* group, and the ATB + Wolfberry+*L. acidophilus* group. The ATB group received a daily gavage of ultrapure water (0.2 mL/d). Meanwhile, the ATB + Wolfberry, ATB + *L. acidophilus*, and ATB + Wolfberry+*L. acidophilus* groups were administered wolfberry decoction (1.75 g/kg/d), *L. acidophilus* (1 × 10^8^ CFU/mouse), and a combination of wolfberry (1.75 g/kg/d) and *L. acidophilus* (1 × 10^8^ CFU/mouse), respectively, via gavage. The entire intervention period lasted for 21 days. The dosages of the above therapeutic drugs were determined based on the body surface area equivalent dose conversion method between mice and humans.

### 2.4 Sperm quality analysis

Euthanasia of mice was performed by cervical dislocation. Following dissection, the epididymis was isolated from each mouse and placed in a Petri dish, and 1 mL of Ham’s F-10 medium (PM151110, Pricella, China) preheated in a 37°C water bath was added. The epididymis was immediately fragmented thoroughly using a syringe needle and incubated at 37°C for 5 min. The coverslip was precisely placed over the counting chamber of the hemocytometer, and the sample was slowly pipetted along the edge of the coverslip to ensure uniform filling of the chamber via capillary action. Using a 40 × objective lens, sperm counts were performed in five central medium squares (each containing 16 small squares) within the large central square of the hemocytometer, following the “count upper but not lower, count left but not right” rule to avoid duplicate counting. Five distinct fields of view were randomly selected, and at least 200 sperm were counted per field. Sperm motility was determined by calculating the percentage of progressively motile sperm (PR) plus non-progressively motile sperm (NP). All procedures were conducted in strict accordance with the standardized protocols outlined in the *WHO laboratory manual for the Examination and processing of human semen (5th Edition)* (World Health Organization, 2010).

### 2.5 Hematoxylin and eosin staining

After the experiment was terminated, the testes and colon tissues of the mice were immediately collected and fixed in 4% paraformaldehyde (BL539A, Biosharp, China) for 24 h. After dehydration with graded ethanol, the tissues were embedded in paraffin, and 4 μm-thick serial sections were prepared (RM2265, Leica, Germany). The sections were baked at 60°C for 2 h. Next, the sections were stained with hematoxylin (BA4097, BaSO, China) and eosin (BA4098, BaSO, China) according to the kit instructions. After differentiation for 4 s, the sections were dehydrated with graded ethanol, mounted with neutral gum, and then observed and imaged using an upright microscope (DM4B, Leica, Germany) (Zhang et al., 2023).

### 2.6 Serum testosterone, follicle-stimulating hormone, and luteinizing hormone concentration determinations

Serum samples from mice were collected and kept at −80°C for future analysis. Following the guidelines of the testosterone (T) detection kit (ml001948, mlbio, China), follicle-stimulating hormone (FSH) detection kit (ml001910, mlbio, China), and luteinizing hormone (LH) detection kit (ml001984, mlbio, China), samples, standards, and HRP-conjugated detection antibodies were added sequentially to a 96-well plate. The plate was sealed, incubated, washed, and developed for colorimetric analysis. Absorbance was measured at 450 nm using a microplate reader (SpectraMax Mini, United States). A standard curve was generated to allow the determination of the concentrations of T, FSH, and LH.

### 2.7 16S rRNA sequencing

Fecal samples collected from mice underwent total microbial DNA extraction and subsequent analysis by Shanghai Personalbio Technology Co., Ltd. Sequencing was performed on the hypervariable V3-V4 region of the bacterial 16S rRNA gene. Following PCR amplification, library construction, library quality control, and library sequencing, the obtained microbiome data were analyzed using QIIME2 version 2022.11 in accordance with the official guidelines.

### 2.8 Transcriptome sequencing and analysis

Testicular tissues were harvested from four randomly selected mice from the normal group, model group, and wolfberry group. Total RNA extraction was entrusted to Shanghai Personalbio Technology Co., Ltd. A cDNA library was constructed via PCR amplification and subjected to quality control. Sequencing of the cDNA library generated raw data, which were filtered to remove adapter sequences and low-quality reads, resulting in clean data for subsequent analyses. The clean reads were aligned to the reference genome using HISAT2 (v2.1.0), and HTSeq (v0.9.1) was employed to calculate the Read Count values for each gene, representing their raw expression levels. Expression levels were normalized using FPKM (Fragments Per Kilobase per Million)/TPM (Transcripts Per Million). Differential expression analysis was performed using DESeq2 (v1.38.3), with the criteria for identifying differentially expressed genes (DEGs) set as |log_2_FoldChange| > 1 and *p*-value < 0.05. A Venn diagram was generated to identify key DEGs. Gene Ontology (GO) and Kyoto Encyclopedia of Genes and Genomes (KEGG) pathway analyses were conducted using clusterProfiler (v4.6.0), with statistical significance defined as a *p*-value < 0.05.

### 2.9 Real-time PCR

Genomic DNA was extracted from mouse fecal samples using a previously established protocol (Lu et al., 2024). Quantitative real-time PCR was performed using an 8-tube PCR system with primers specific for *L. acidophilus* (forward primer: 5′-AGCAGTAGGGAATCTTCCA-3′, reverse primer: 5′-CACCGCTACACATGGAG-3′), total bacteria (forward primer: 5′-ACTCCTACGGGAGGCAGCAGT-3′, reverse primer: 5′-ATTACCGCGGCTGCTGGC-3′), ddH₂O, and SuperReal PreMix Plus (FP205-02, TIANGEN, China). Amplification was performed on a real-time fluorescence quantitative PCR instrument (Bio-Rad, United States). Data analysis was conducted based on the cycle threshold (CT) values, employing the 2^−ΔΔCT^ method for processing and quantifying the results.

### 2.10 Periodic acid-Schiff staining

Glycoconjugates in 4 μm-thick paraffin-embedded colon sections were stained using a Periodic acid-Schiff staining (PAS) kit (R20526, Yuanye, Shanghai). The staining procedure included: dewaxing in xylene, rehydration with graded ethanol, oxidation with 0.5% periodic acid, staining with Schiff reagent in the dark, counter-staining with hematoxylin, and mounting with neutral resin (Lu et al., 2024). The stained sections were imaged using a light microscope system (DM4B, Leica, Germany), and the goblet cells and crypt depth were quantitatively analyzed using ImageJ software.

### 2.11 Immunohistochemistry

After antigen retrieval, colonic tissue sections were stained using an immunohistochemistry kit (PV-9000, ZSGB-BIO, China), according to the manufacturer’s instructions. Sections were incubated overnight at 4°C with a rabbit monoclonal primary antibody specific to Zonula occludens-1 (ZO-1) tight junction protein (ab276131, Abcam, Cambridge, UK) at a dilution of 1:300. Subsequently, a 3,3′-diaminobenzidine (DAB) chromogenic substrate solution was applied using a DAB kit (ZLI-9018, ZSGB-BIO, China). After counterstaining with hematoxylin, the sections were dehydrated, cleared, and mounted (Lu et al., 2023). Microscopic observation and photography were performed using a microscope (DM4B, Leica, Germany), followed by quantitative analysis utilizing ImageJ software.

### 2.12 Statistical analysis

Data analysis and visualization were executed using GraphPad Prism 8.0 software. To compare multiple groups of mice, one-way ANOVA was utilized, followed by Tukey’s test for individual group comparisons. For evaluating overall differences in α-diversity among groups, the Kruskal-Wallis nonparametric test was applied, with Dunn’s test used for *post-hoc* pairwise comparisons. Statistical significance was determined as a *p*-value lower than 0.05.

## 3 Results

### 3.1 Wolfberry protected against reproductive injury induced by anti-TB drugs in male mice

**Figure 1:**
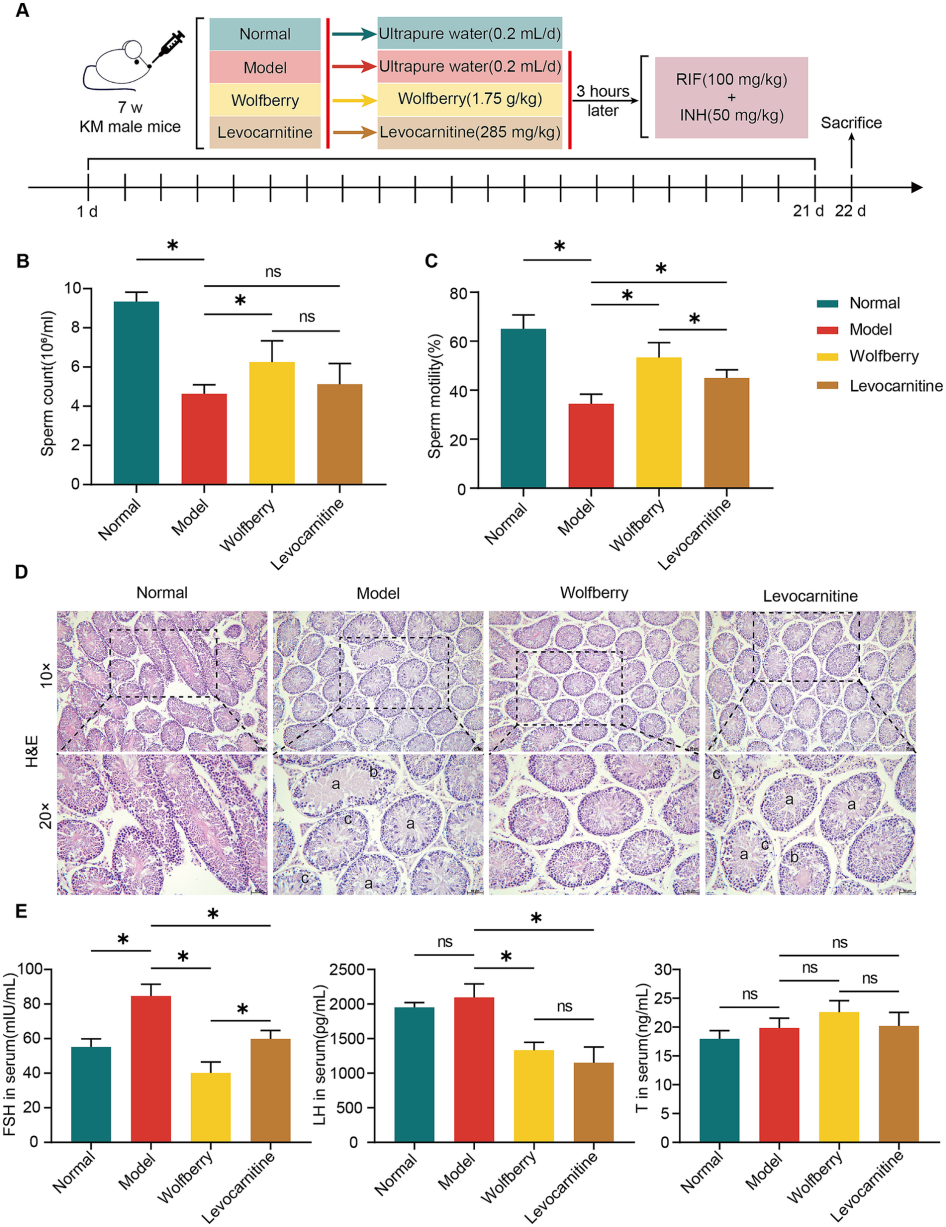
Wolfberry protected against reproductive injury induced by anti-TB drugs in male mice. **(A)** Schematic diagram of the grouping and treatment of mice. **(B)** Mice sperm count. **(C)** Mice sperm motility. **(D)** Testicular histopathology was observed using H&E (scale = 50 μm, a indicates sperm cells and sperm shedding, b shows a disordered arrangement of spermatogenic cells, c indicates a decrease in spermatogonia, primary spermatocytes and secondary spermatocytes). **(E)** Levels of FSH, LH, and T in mice serum. Data are represented as mean ± SD. *p < 0.05.

After the model was established and interventions were completed, we assessed the sperm counts in each group of mice (Figure 1A). The results revealed that the sperm count in the model group was reduced by 50.38% compared to the normal group. Compared with the model group, the wolfberry group exhibited a 34.99% increase in sperm count, whereas the levocarnitine group showed some improvement, though it did not reach statistical significance (Figure 1B). Clinically, levocarnitine has been proven to significantly improve sperm motility and is commonly used in the treatment of male reproductive injuries (Tang et al., 2008; Liu et al., 2025). Based on this, it was used as a positive control drug in this study. Analysis of sperm motility revealed a reduction of 47.02% in the model group relative to the normal group. Compared to the model group, the wolfberry group exhibited a 54.85% enhancement in sperm motility, and the levocarnitine group showed a 30.66% improvement. Importantly, the increase in sperm motility in the wolfberry group was markedly superior to that in the levocarnitine group (Figure 1C).

Hematoxylin and eosin (H&E) staining of testicular tissues revealed significant pathological differences among mouse groups. The model group exhibited thinned spermatogenic epithelium, disordered arrangement of spermatogenic cells, reduced cell layers, and absence of sperm in the lumen. In contrast, the wolfberry group showed thickened spermatogenic epithelium, increased number of spermatogenic cells, and well-organized testicular tissue structure. No obvious improvement in testicular pathological changes was observed in the levocarnitine group (Figure 1D).

Furthermore, the serum levels of sex hormones were assessed. The FSH levels in the model group were significantly elevated compared to the normal group. In contrast, the wolfberry group showed significantly lower FSH levels than the model group, while no significant changes were noted in the levocarnitine group. For LH levels, the model group displayed significantly higher levels compared to both the wolfberry and levocarnitine groups, whereas no significant differences were observed between the normal group and the other groups. There were no significant differences in T levels among the groups (Figure 1E). Overall, these results suggested that wolfberry protects against reproductive injuries induced by anti-TB drugs in male mice.

### 3.2 Wolfberry regulated gut dysbiosis induced by anti-TB drugs in mice

**Figure 2:**
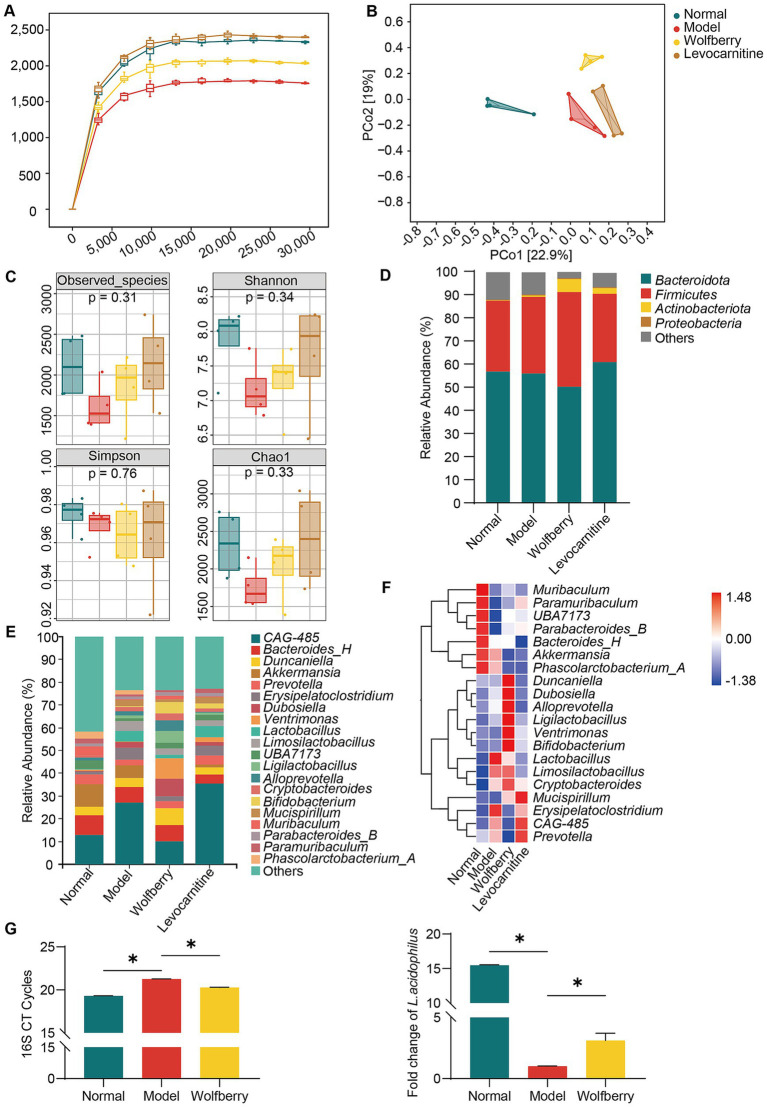
Wolfberry regulated gut dysbiosis induced by anti-TB drugs in mice. **(A)** The sparse curve of 16S rRNA gene sequencing in mice from different groups. **(B)** The PCoA diagram of mice from different groups. **(C)** The alpha diversity indices (Observed_species, Shannon, Simpson, Chao1) of mice from different groups. **(D)** Composition of microflora (top 4) at the phylum level in each group of mice. **(E)** Composition of microflora (top 20) at the genus level in each group of mice. **(F)** Heat map displaying the top 20 species at the genus level in each group of mice. **(G)** Following the intervention, changes in the abundance of total bacteria and *L. acidophilus* among experimental mouse groups were analyzed by real-time PCR. Data are represented as mean ± SD. *p < 0.05.

To investigate the GM changes associated with the protective effects of wolfberry against reproductive injury induced by anti-TB drugs, 16S rRNA sequencing was performed on mouse fecal samples. As the sequencing depth increased, the rarefaction curve plateaued, indicating that the current sequencing depth adequately captured most species in the samples, and the data met the requirements for species diversity analysis (Figure 2A). Principal coordinate analysis (PCoA) based on the Bray-Curtis algorithm was conducted to assess similarities and differences in GM among groups. Significant differences in microbiota composition were observed among the normal, model, wolfberry, and levocarnitine groups (Figure 2B). To further evaluate α-diversity within the microbial communities, indices such as Observed_species, Chao1, Shannon, and Simpson were analyzed. The results revealed a reduction in species richness and diversity in the model group, while these metrics increased in the wolfberry group (Figure 2C).

Next, we analyzed the relative abundances of microbial taxa at various taxonomic levels to identify characteristic microorganisms associated with different stages. At the phylum level, *Firmicutes* and *Bacteroidetes* comprised over 80% of the total microbiota. The ratios of *Firmicutes* to *Bacteroidetes* in the normal, model, wolfberry, and levocarnitine groups were 0.54, 0.59, 0.82, and 0.48, respectively, with the wolfberry group showing a notable increase in *Firmicutes* abundance (Figure 2D). At the genus level, compared to the normal group, the model group displayed reduced proportions of *Paramuribaculum* and *Akkermansia*, along with an increased proportion of *Erysipelatoclostridium*. Conversely, the wolfberry group exhibited higher proportions of *Bifidobacterium* and *Ligilactobacillus* and a lower proportion of *Erysipelatoclostridium* relative to the model group (Figures 2E,F). These observations indicated that wolfberry effectively mitigated gut dysbiosis caused by anti-TB drugs. Moreover, real-time PCR was employed to assess changes in total bacterial load and *L. acidophilus* abundance among the groups. Compared to the normal group, the model group exhibited a reduced total bacterial count. However, the wolfberry group showed a significant increase in total bacterial count compared to the model group. Besides, while the abundance of *L. acidophilus* significantly decreased in the model group compared to the normal group, the wolfberry group exhibited a 3.14-fold increase in abundance compared to the model group (Figure 2G). These findings collectively highlight that wolfberry markedly elevated the abundance of *L. acidophilus* in the mouse gut.

### 3.3 Wolfberry mitigated anti-TB drug-induced damage to the gut barrier in mice

**Figure 3:**
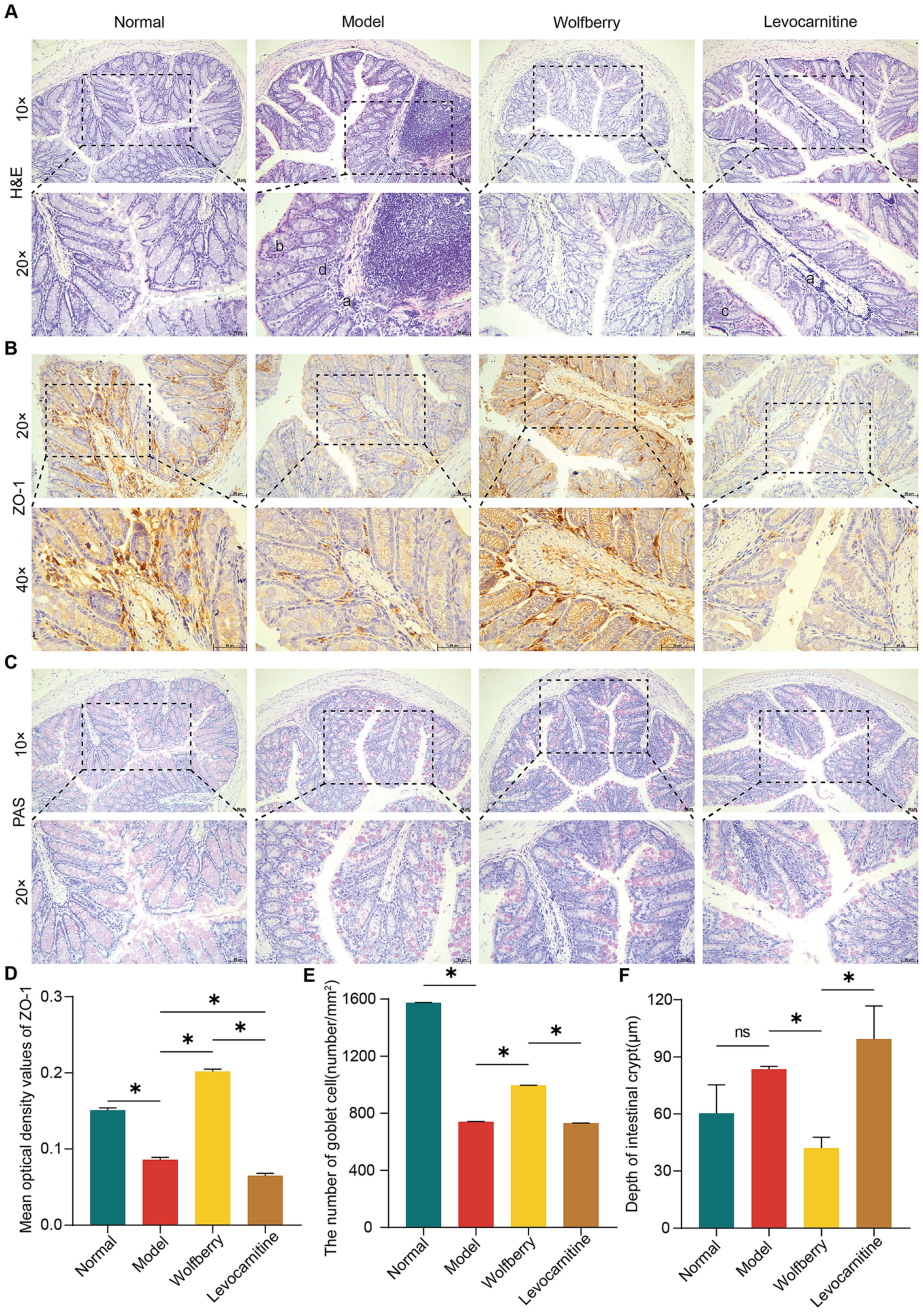
Wolfberry mitigated anti-TB drug-induced damage to the gut barrier in mice. **(A)** Colonic histopathology was observed using H&E staining (scale = 50 μm, a exhibiting infiltration of inflammatory cells in the mucosal layer of the colon, b illustrating the uneven arrangement of epithelial cells in the mucosal layer of the colon, c representing abnormal villi arrangement, d showing the disrupted intestinal gland structure). **(B)** IHC staining of mouse colon for ZO-1 expression (scale = 50 μm). **(C)** The number of goblet cells in the colon of mice was observed using PAS staining (scale = 50 μm). **(D)** Quantification of ZO-1 expression in mouse colon. **(E)** The number of colon goblet cells in each group. **(F)** Colon crypt depth in mice in each group. Data are represented as mean ± SD. *p < 0.05.

The impairment of the gut barrier, characterized by enhanced gut permeability, is a typical manifestation of gut dysbiosis (Di Vincenzo et al., 2024). Such barrier dysfunction has been linked to dysregulated spermatogenesis (Wang and Xie, 2022; Lv et al., 2024). To assess the gut barrier function, we compared H&E-stained colon sections from mice across different groups. Our observations indicated that, relative to the normal group, the model group exhibited disorganized epithelial cell alignment in the colonic mucosa, accompanied by inflammatory cell infiltration and damage to the intestinal gland architecture. The levocarnitine group also showed pathological changes, such as inflammatory cell infiltration and disorganized villi arrangement. Conversely, the wolfberry group demonstrated a healthy intestinal structure, with no significant signs of structural damage or inflammation (Figure 3A).

ZO-1 plays a vital role in preserving the gut barrier’s function, with goblet cells being responsible for the formation of the protective mucus layer in the colon. Moreover, the depth of the crypts can be associated with the presence of toxins, providing insights into the overall health of the intestine (Jian et al., 2021; Nyström et al., 2021; Kpomasse et al., 2023). In the present study, we employed Immunohistochemistry (IHC) to evaluate ZO-1 expression, PAS staining for goblet cell counts, and measured crypt depths in the colon. Compared to the normal group, the model group exhibited decreased ZO-1 expression, fewer goblet cells, and increased crypt depth, indicating compromised gut barrier function due to anti-TB drugs. In contrast, the wolfberry group showed improved gut health with higher ZO-1 expression, shallower crypts, and an increased number of goblet cells, effectively mitigating gut damage observed in the model group. No significant differences were noted between the levocarnitine and model groups in terms of gut pathology, ZO-1 levels, crypt depth, or goblet cell counts (Figures 3C–F). Our findings collectively indicated that wolfberry enhances gut barrier function in mice suffering from reproductive injuries induced by anti-TB drugs.

### 3.4 Wolfberry protected against reproductive injury induced by anti-TB drugs by regulating spermatid differentiation, promoting spermatid development, and modulating the meiotic cell cycle

**Figure 4:**
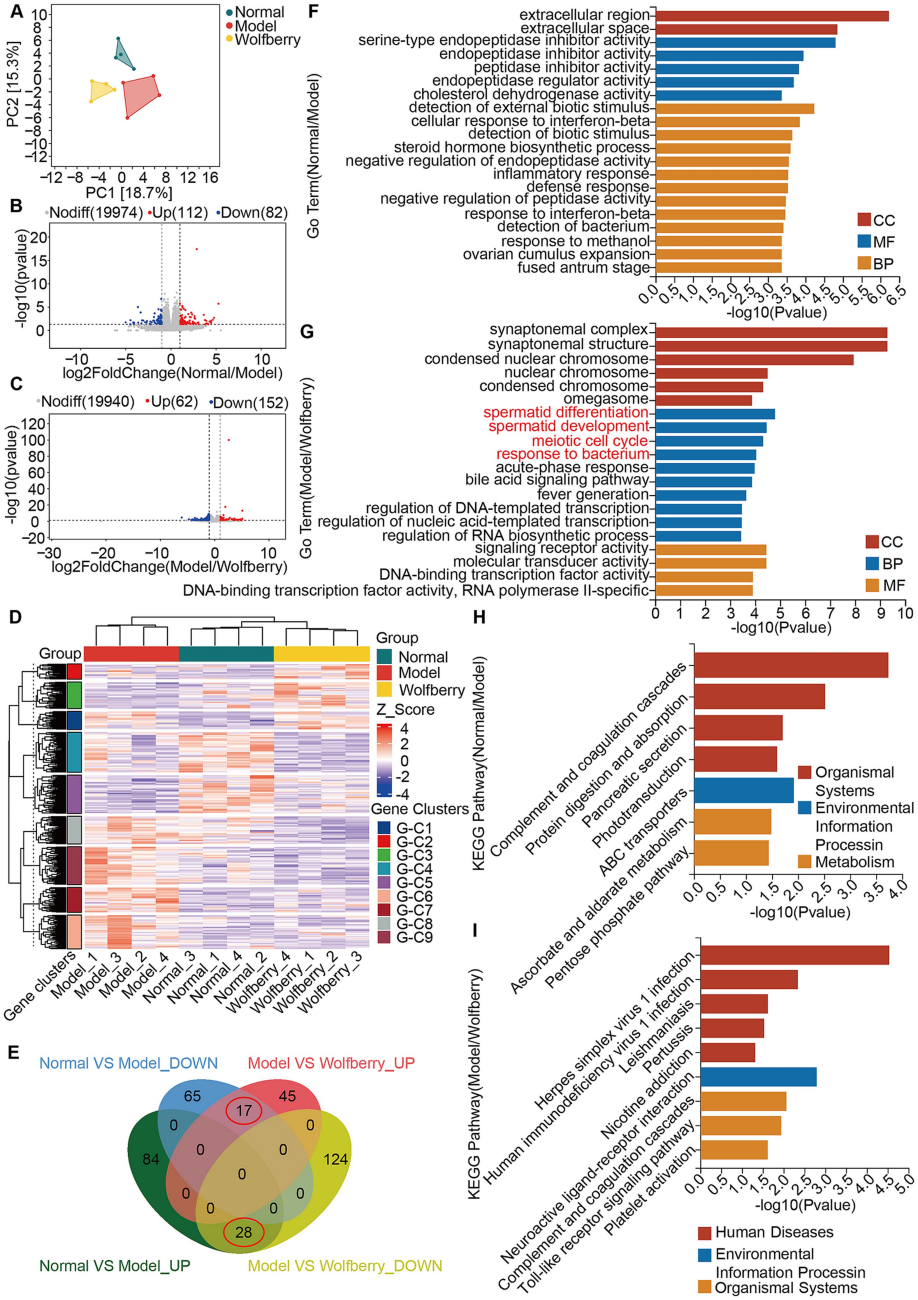
Wolfberry protected against reproductive injury induced by anti-TB drugs by regulating spermatid differentiation, promoting spermatid development, and modulating the meiotic cell cycle. **(A)** Principal component analysis. **(B)** Volcano plot of DEGs between the Normal group vs. the Model group. **(C)** Volcano plot of DEGs between the Model group vs. the Wolfberry group. **(D)** Heatmap clustering of DEGs. **(E)** Venn diagram of intersecting DEGs. **(F)** GO analysis between the Normal group vs. the Model group. **(G)** GO analysis between the Model group vs. the Wolfberry group. **(H)** KEGG analysis between the Normal group vs. the Model group. **(I)** KEGG analysis between the Model group and Wolfberry group.

Principal component analysis of the testicular transcriptome data highlighted significant distinctions among the groups (Figure 4A). Using criteria of |log_2_FC| > 1 and a *p*-value < 0.05, we identified 465 DEGs. Volcano plots constructed based on fold change (FC) and *p*-values illustrated the significant gene expression differences between the normal and model groups, as well as between the model and wolfberry groups. Specifically, there were 194 DEGs between the normal and model groups (112 upregulated and 82 downregulated), and 214 DEGs between the model and wolfberry groups (62 upregulated and 152 downregulated) (Figures 4B,C). Heatmap clustering analysis further demonstrated clear distinctions in DEG expression among groups, qualifying them for further analysis (Figure 4D). We identified 45 common DEGs between the comparisons of normal vs. model and model vs. wolfberry groups, prompting their selection as key targets for this study. A Venn plot revealed that 28 DEGs upregulated in the model group were downregulated following wolfberry treatment, while 17 DEGs downregulated in the model group were upregulated after wolfberry intervention (Figure 4E). Consequently, these 45 DEGs were considered potential targets for treating anti-TB drug-induced reproductive injuries with wolfberry and were used in subsequent functional enrichment analyses.

GO and KEGG pathway analyses were conducted for both normal vs. model and model vs. wolfberry group comparisons (Figures 4F–I). Notably, GO analysis between the model and wolfberry groups highlighted four prominent terms within the Cellular Component (CC) category: spermatid differentiation, spermatid development, meiotic cell cycle, and response to bacterium (Figure 4G). These findings suggest that wolfberry not only affects genes involved in spermatogenesis but also plays a role in regulating the GM.

### 3.5 Wolfberry exerted its effects independent of the GM and demonstrated a synergistic effect with *Lactobacillus acidophilus*

**Figure 5:**
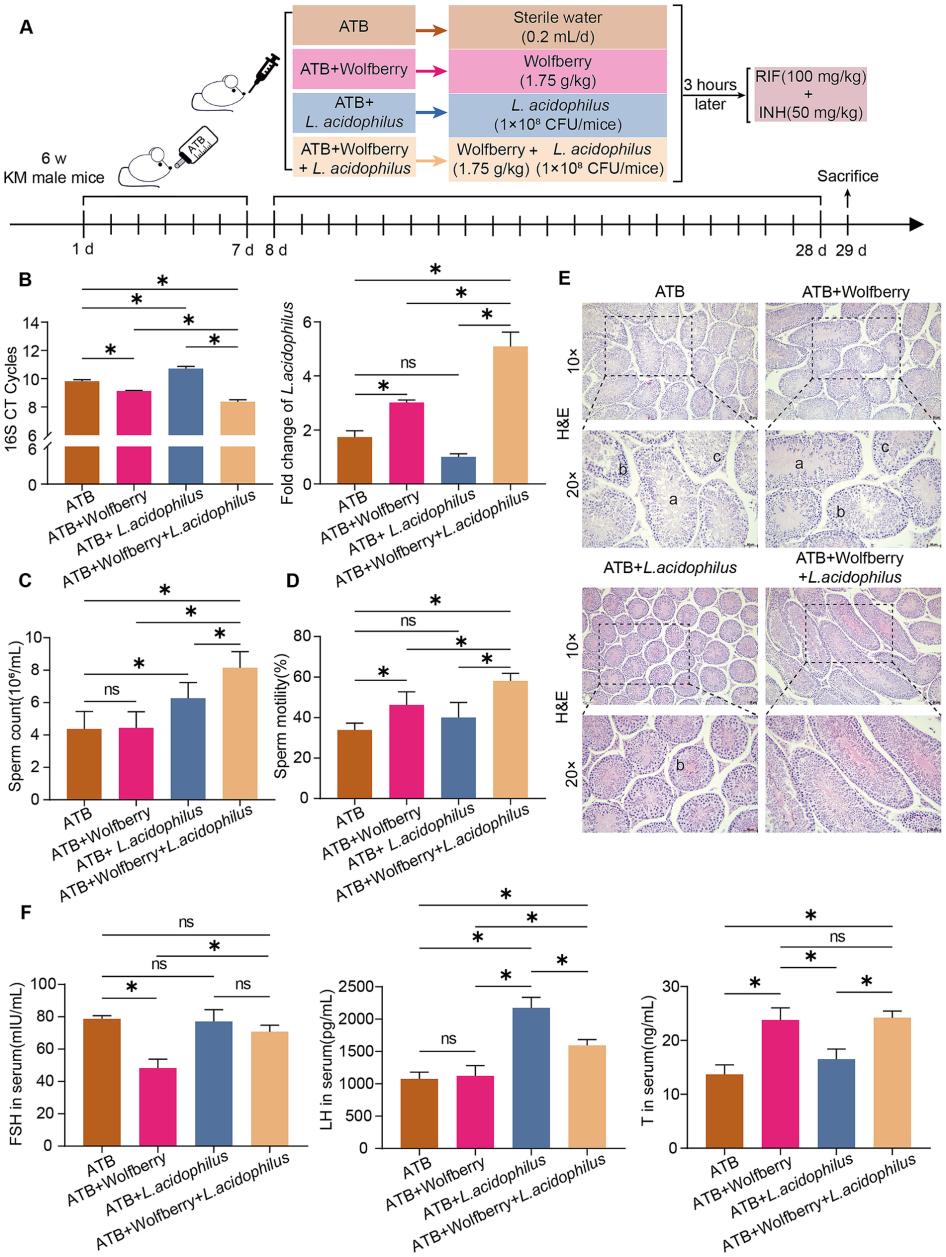
Wolfberry exerted its effects independent of the GM and demonstrated a synergistic effect with *L. acidophilus*. **(A)** Schematic diagram of the establishment of pseudo-germ-free mice, their subsequent grouping, and the intervention strategies employed. **(B)** The abundance of total bacteria and *L. acidophilus* in the intestinal tract of mice from each experimental group was quantified by real-time PCR. **(C)** Mice sperm count. **(D)** Mice sperm motility. **(E)** Testicular histopathology was observed using H&E (scale = 50 μm, a indicates sperm cells and sperm shedding, b shows a disordered arrangement of spermatogenic cells, c indicates a decrease in spermatogonia, primary spermatocytes and secondary spermatocytes). **(F)** Levels of FSH, LH, and T in mouse serum. Data are represented as mean ± SD. *p < 0.05.

To investigate whether GM mediates the protective effects of wolfberry against reproductive injury caused by anti-TB drugs, we established a pseudo-germ-free mice model using ATB water and subsequently administered wolfberry to the mice (Figure 5A). Compared with the wolfberry group, the ATB + wolfberry group showed significantly lower sperm count and motility, and pathological changes in testicular tissues, including thinned spermatogenic epithelium, disordered arrangement of spermatogenic cells, and absence of sperm in the lumen. These findings indicated that GM plays a critical role in the protective mechanism of wolfberry.

As shown in Figure 2G, wolfberry significantly increased the abundance of *L. acidophilus* in the mouse gut. Based on this, we supplemented wolfberry administration with *L. acidophilus* gavage to further explore their combined effects. The results showed that compared to the ATB + Wolfberry group, the ATB + Wolfberry+*L. acidophilus* group displayed significantly improved sperm count and motility, restored testicular damage, and elevated levels of FSH and LH. Compared with the ATB group, the ATB + wolfberry group showed no significant improvement in sperm motility or testicular pathology. Both groups exhibited pathological changes in testicular tissues, including thinned spermatogenic epithelium, disordered arrangement of spermatogenic cells, and absence of sperm in the lumen, although sperm motility and testosterone levels were significantly higher in the latter group (Figures 5C–F).

Real-time PCR analysis of *L. acidophilus* abundance in the gut revealed that both the ATB + Wolfberry and ATB + Wolfberry+*L. acidophilus* groups exhibited increased *L. acidophilus* levels compared to the ATB group. Notably, the ATB + Wolfberry+*L. acidophilus* group exhibited significantly higher *L. acidophilus* abundance than the ATB + Wolfberry group. These findings suggest that wolfberry and *L. acidophilus* exert synergistic effects, though wolfberry’s protective mechanisms extend beyond GM modulation. Furthermore, compared with the ATB + *L. acidophilus* group, the ATB + wolfberry+*L. acidophilus* group showed significant improvements in sperm count and motility, increased *L. acidophilus* abundance, elevated testosterone levels, and testicular tissues with thickened spermatogenic epithelium, orderly arrangement of spermatogenic cells, increased cell layers, and increased sperm count in the lumen (Figures 5B–F). Collectively, these results underscore the synergistic role of wolfberry and *L. acidophilus* in protecting against anti-TB drug-induced reproductive injury.

### 3.6 The combination of wolfberry and *Lactobacillus acidophilus* more effectively restored anti-TB drug-induced intestinal barrier dysfunction in mice compared to either treatment alone

**Figure 6:**
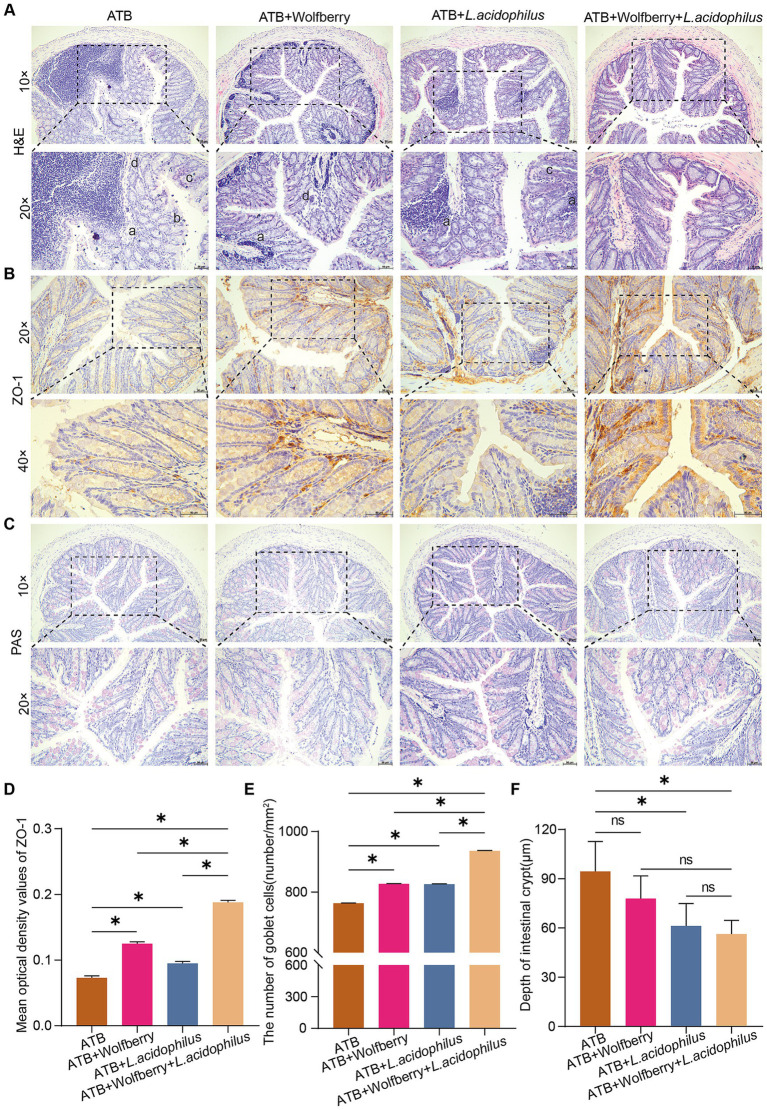
The combination of wolfberry and *L. acidophilus* more effectively restored anti-TB drug-induced intestinal barrier dysfunction in mice compared to either treatment alone. **(A)** Colonic histopathology was observed using H&E staining (scale bar = 50 μm, a infiltration of inflammatory cells in the mucosal layer of the colon, b uneven arrangement of epithelial cells in the mucosal layer of the colon, c abnormal villi arrangement, d disrupted intestinal gland structure). **(B)** IHC staining of mouse colon for ZO-1 expression (scale = 50 μm). **(C)** The number of goblet cells in the colon of mice was observed using PAS staining (scale = 50 μm). **(D)** Quantification of ZO-1 expression in mice colon. **(E)** The number of colon goblet cells in each group. **(F)** Colon crypt depth in mice in each group. Data are represented as mean ± SD. *p < 0.05.

We further investigated the impact of combining wolfberry with *L. acidophilus* on intestinal barrier function. Pathological alterations in the colons of mice from each group were examined via H&E staining. The findings revealed that the ATB, ATB + Wolfberry, and ATB + *L. acidophilus* groups exhibited damage, such as inflammatory cell infiltration in the colonic mucosa, disordered epithelial cell alignment, irregular villi arrangement, and structural damage to intestinal glands. In contrast, H&E staining of the ATB + Wolfberry+*L. acidophilus* group indicated intact colonic tissue structure, orderly arranged epithelial cells in the mucosal layer, and evenly distributed goblet cells (Figure 6A). IHC analysis was employed to assess ZO-1 expression levels in each group’s colons. Results demonstrated that compared to the ATB group, ZO-1 expression increased in the ATB + Wolfberry, ATB + *L. acidophilus*, and ATB + Wolfberry+*L. acidophilus* groups, with the ATB + Wolfberry+*L. acidophilus* group showing notably higher ZO-1 expression than both the ATB + Wolfberry and ATB + *L. acidophilus* groups (Figures 6B,D). Subsequent PAS staining and crypt depth measurements revealed that the ATB + Wolfberry+*L. acidophilus* group exhibited the highest number of goblet cells and the shallowest crypt depth (Figures 6C,E,F). These outcomes suggest that the combination of wolfberry and *L. acidophilus* can effectively repair anti-TB drug-induced intestinal structural damage in mice and enhance intestinal barrier function.

## 4 Discussion

**Figure 7:**
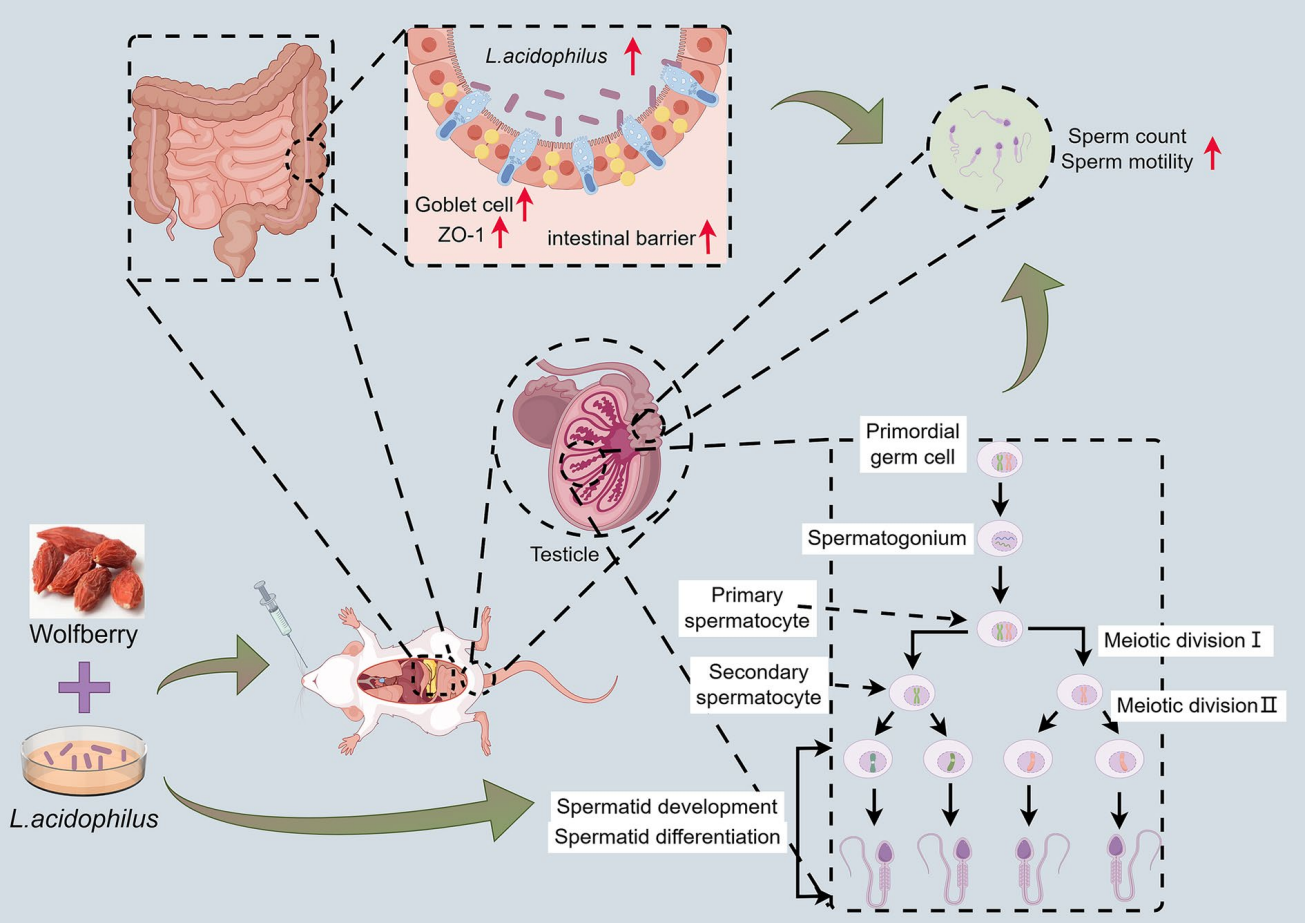
Wolfberry worked synergistically with *L. acidophilus* to protect against reproductive injury induced by anti-TB drugs.

In recent years, the increasing global burden of TB has raised significant concerns about its impact on public health, especially regarding the side effects of anti-TB drugs (Ryu et al., 2025; Lin et al., 2025). Our study explored the protective effect of wolfberry, a traditional medicinal and edible plant, on male reproductive injury induced by anti-TB drugs. Our findings indicated that wolfberry could alleviate reproductive damage through multiple mechanisms, particularly in regulating GM and promoting the abundance of beneficial bacteria such as *L. acidophilus* (Figure 7). Notably, the protective effect of wolfberry against anti-TB drug-induced reproductive damage is not entirely dependent on GM modulation.

The GM is increasingly acknowledged for its crucial role in regulating the host’s health, encompassing reproductive functions (Lv et al., 2024). Prior research has indicated that disruptions in the GM can result in changes to steroid hormone levels, sperm production, and testicular function (Ding et al., 2020; Wang and Xie, 2022; Ashonibare et al., 2024). Herein, we found that administering RIF and INH to mice induced gut dysbiosis characterized by a decrease in beneficial bacteria like *Paramuribaculum* and *Akkermansia*. These beneficial bacteria are associated with reduced intestinal permeability and protection of the mucosal barrier (Xu et al., 2023; Mo et al., 2024; Yang and Zhong, 2025). Besides, our results showed that the addition of wolfberry enhanced the abundance of *Bifidobacterium* and *Ligilactobacillus*. These two probiotic strains contribute positively to intestinal health and the maintenance of the intestinal barrier (Schroeder et al., 2018; Mao et al., 2024; Gu et al., 2025).

Moreover, our study highlighted the significant role of gut microbiota in regulating spermatogenesis. The testis, being a highly specialized organ for germ cell development, is particularly sensitive to alterations in the GM. A disrupted GM has been shown to impair testicular function, reduce sperm count, and disturb spermatogenesis (Wang and Xie, 2022; Zhang et al., 2025; Wu et al., 2025). Our findings corroborated these observations, as the administration of anti-TB drugs led to pathological changes in the testis, including decreased spermatogenic activity and testicular tissue damage. The GM’s influence on androgen metabolism is also an important factor to consider. Studies have shown that the GM can regulate androgen production, with specific gut bacteria influencing testosterone levels (Colldén et al., 2019). In this research, the addition of wolfberry enhanced the abundance of *L. acidophilus* and raised serum T levels, further reinforcing the concept that the GM can be utilized to safeguard male reproductive health.

Interestingly, the combination of wolfberry and *L. acidophilus* resulted in a synergistic effect in protecting against reproductive injury induced by anti-TB drugs. *L. acidophilus*, a well-known probiotic, has been reported to enhance intestinal barrier function, reduce inflammation, and restore gut health (Anderson et al., 2010; Wu et al., 2020; Tegegne and Kebede, 2022). In addition, *L. acidophilus* exerts a protective effect on the testes and epididymis of rats (Amdekar and Singh, 2016; Sheikh Hosseini et al., 2019). In our study, the addition of *L. acidophilus* further improved the effects of wolfberry, suggesting a cooperative mechanism in optimizing both gut health and male reproductive health. This synergistic effect highlights the potential therapeutic value of combining natural substances to enhance the overall health of individuals undergoing TB treatment.

## 5 Conclusion

In summary, our research offers compelling evidence that the preservation of male reproductive health by wolfberry during TB therapy is associated with GM regulation and enhancement of intestinal barrier function. Furthermore, the combination of wolfberry and *L. acidophilus* represents a promising strategy for mitigating the reproductive toxicity of anti-TB drugs. Overall, our findings not only underscore the importance of the GM in reproductive health but also introduce a novel approach to protect male fertility during TB treatment.

## Data Availability

The original contributions presented in the study are publicly available. The transcriptomic and 16S rRNA sequencing data can be found in the NCBI repositories through accession number: PRJNA1286912, https://www.ncbi.nlm.nih.gov/bioproject/PRJNA1286912 and accession number: PRJNA1286962, https://www.ncbi.nlm.nih.gov/bioproject/PRJNA1286962.
